# DoE-derived continuous and robust process for manufacturing of pharmaceutical-grade wide-range LNPs for RNA-vaccine/drug delivery

**DOI:** 10.1038/s41598-022-12100-z

**Published:** 2022-06-07

**Authors:** Kakon Nag, Md. Enamul Haq Sarker, Samir Kumar, Habiba Khan, Sourav Chakraborty, Md. Jikrul Islam, Juwel Chandra Baray, Maksudur Rahman Khan, Asif Mahmud, Uttam Barman, Eleus Hussain Bhuiya, Mohammad Mohiuddin, Naznin Sultana

**Affiliations:** Globe Biotech Limited, 3/Ka (New) Tejgaon I/A, Dhaka, 1208 Bangladesh

**Keywords:** Biotechnology, Nanoscience and technology

## Abstract

Lipid nanoparticle (LNP) technology has become extremely demanding for delivering RNA-products and other drugs. However, there is no platform to manufacture pharmaceutical-grade LNPs with desired particle size from a wide range in continuous mode. We have developed a unique platform to obtain any specific size-range of LNPs from 60 to 180 nm satisfying pharmaceutical regulatory requirements for polydispersity index, sterility, dose uniformity and bio-functionality. We applied design of experiment (DoE) methodology and identified the critical process parameters to establish the process for global application. Cross-point validation within the response map of DoE confirmed that the platform is robust to produce specific size (± 10 nm) of LNPs within the design-range. The technology is successfully transformed to production scale and validated. Products from R&D, pilot and production batches for a candidate SARS-CoV-2 mRNA-vaccine generated equivalent biological responses. The data collectively established the robustness and bio-uniformity of doses for global RNA-vaccine/drug formulation.

## Introduction

The RNAs (mRNA, miRNA, shRNA, siRNA, tRNA etc.) are very promising candidates for therapeutics but difficult to deliver into intracellular milieu. Formulation of RNAs through formation of lipid nanoparticle (LNP) overcomes the challenges^1–4^. The selection of lipid composition in proper ratio, LNP formation process, optimization of buffer for RNA sample preparation, stabilization and formulation buffers for the RNA-LNPs collectively play vital roles to control the size and homogeneity of particles^5^.

Different sizes of RNA-LNPs are required for different therapeutics based on target organ or tissue or the desired physiological responses^6–9^. Therefore, US FDA emphasizes on the size and size distribution of particles as “critical quality attributes (CQA)” for liposome drug^10^. The polydispersity index (PDI) value of the LNPs should be ≤ 0.30 to qualify as a drug as per regulatory requirement^11^. Formation and stabilization of RNA-LNPs are crucial because, for most of the cases, sizes and PDI of LNPs get changed during the steps of manufacturing process^12^. Hence, production of LNPs with specific size and PDI according to the regulatory requirement remains a big challenge^13^.

Ostwald ripening is an intrinsic property of LNPs. Small LNPs gradually fuse to form larger LNPs to get better energetically stabilized condition^14^. Natural Ostwald ripening is comparatively a slower process, and it takes long time to grow larger LNPs at rest. In fact, gradual enlargement of LNPs at rest is a challenge for the technology to overcome for achieving longer shelf life of doses^15^. Here, we have invented and established a suitable process to overcome these challenges through DoE approach.

## Results and discussion

**Figure 1:**
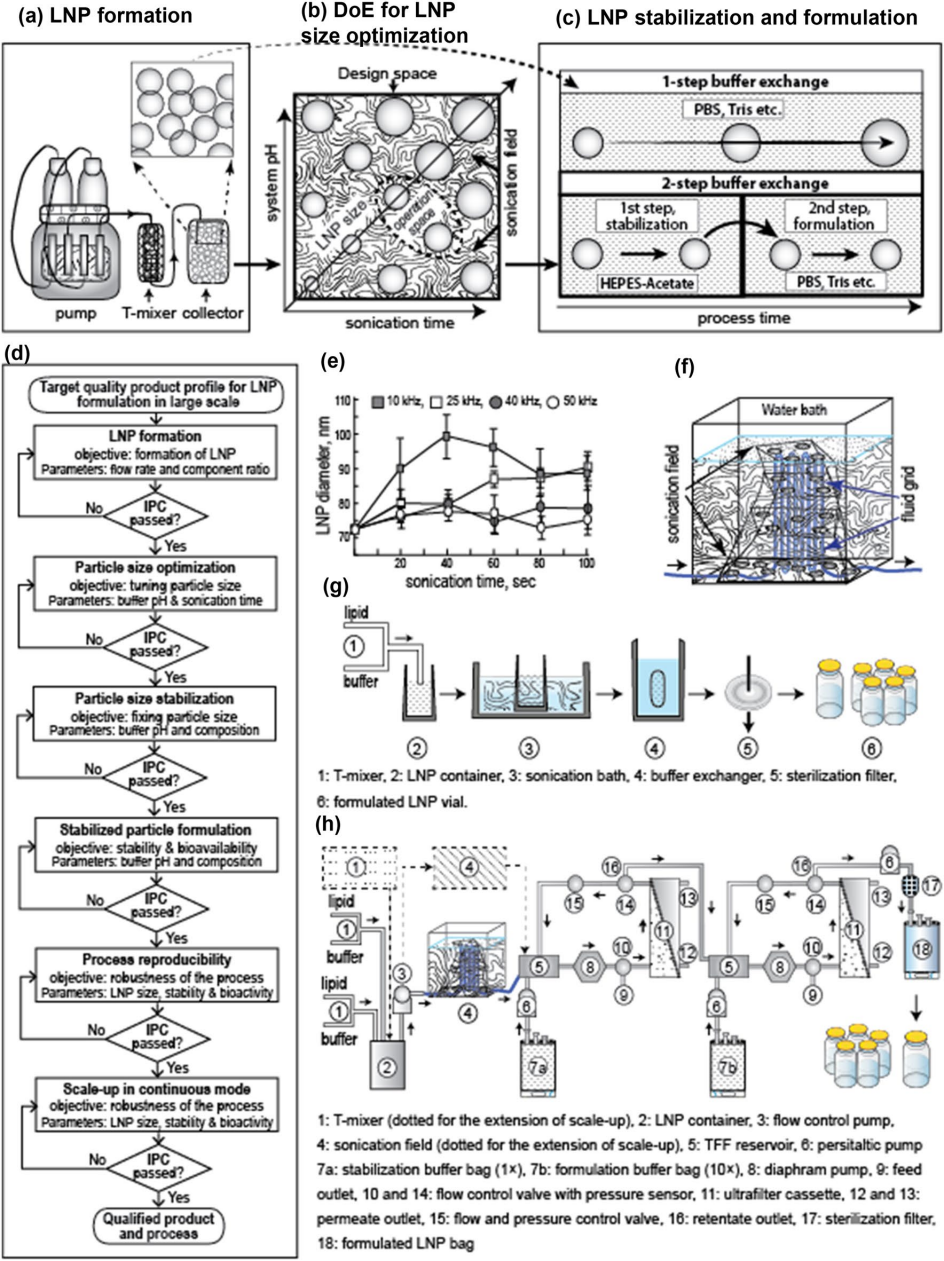
Schematics of the developed process and comparison with existing method. (**a**) LNP is formed using a simple T-mixer serviced by flow-controlled syringe pumps. The process flow is indicated by arrow; dotted arrow indicates zoom view of the LNP size. (**b**) The DoE model and result of the DoE experiments revealed that in a sonication field the size of the LNPs gradually increased with the growing time and pH. Desired size of LNP can be achieved by selecting specific ‘operation space’ (dotted oval). (**c**) Single step buffer exchange is associated with the gradual size enlargement of LNP (top) but a 2-stage buffer exchange restricts LNP size (bottom). (**d**) Process development decision tree. (**e**) Different level of sonication power produced different level of effect on size enlargement of LNP. (**f**) A schematic diagram of relative position of the flow-path of fluid in sonication field of a water bath sonicator. (**g**) Process flow for small volume (1 ml and 10 ml) batches. (**h**) Process flow for large batch in continuous mode.

The technological aspects of the process flow are shown in Fig. 1. We found that after the formation of LNPs, a low-frequency sonication at a specific range in a continuous motion system has provided a suitable kinetics of the organic growth of LNPs in such a manner that the rate can be monitored and utilized to harvest LNPs within a desired size-range (Fig. 1a–c). This invention is based on the preliminary observation that different level of sonication has varying effects on LNP size (Fig. 1e), and it can be reproduced if the sonication field is properly identified (Fig. 1f). We have designed a decision tree based on this observation and proceeded for detail characterization of the findings including scale-up (Fig. 1d); manual process (Fig. 1g) was used for small-scale batch and continuous process (Fig. 1h) was used for large-scale batch.

**Figure 2:**
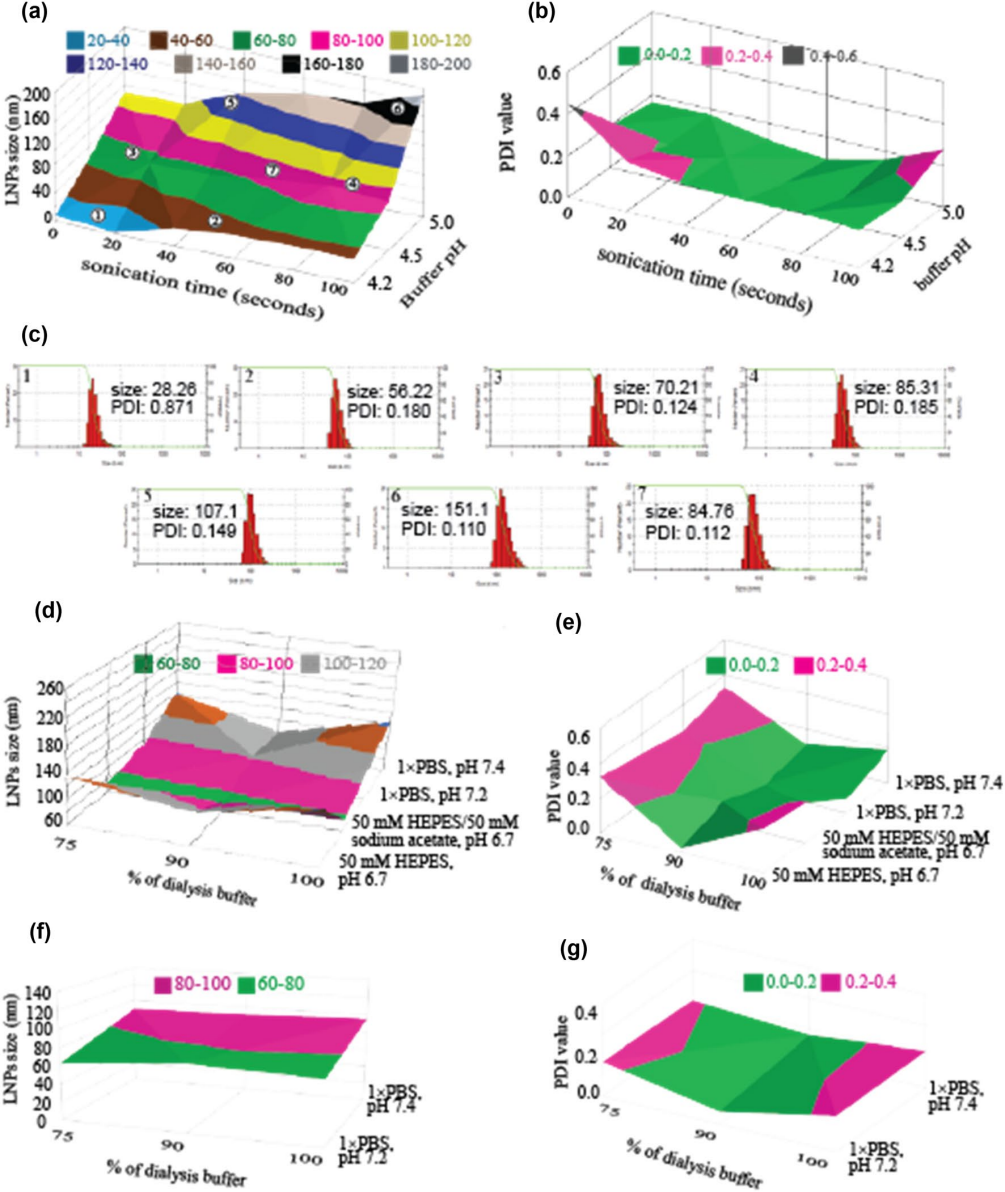
DoE of mRNA-LNPs formation, cross-point validation and buffer change. (**a**) Gradual changes in LNP size in respect of varying time and pH; specific size ranges of LNPs are depicted in different colors. Numerical values in white circle indicate representative conditions for the model validity test. (**b**) Relevant data for PDI values are shown. (**c**) A representative histogram set from triplicate experiments of model validity test is shown after LNP formation. (**d**,**f**) The effect of buffer change on LNP size during stabilization and formulation, and (**e**,**g**) relevant PDI values, respectively. *n* = 3 for all experiments.

This is a novel aspect in LNP formation and processing technology. First, through a simple DoE model (Supplementary Fig. 1a,b), we have obtained a time dependent enlargement of LNPs in different pH and produced a kinetic surface response plot (Fig. 2a, b and Supplementary Fig. 2–4). After obtaining this pH- and time-dependent kinetic map of LNP-growth (Supplementary Table 1 and 2), we have tested the model through performing cross-point validation by choosing seven specific sizes of LNPs covering two extreme diagonals, 4 mid-points around the edges and the center-point of the surface response map (Fig. 2a). The hypothesis was as such that if the kinetic map model is true than one should be able to obtain LNPs within a specific size-range by selecting the combinations of specific time-point and certain pH. The hypothesis was found factual as we were able to harvest different desired sizes of LNPs from different quadrants of the kinetic map-model following the recommended conditions deciphered from the model (Fig. 2c, Supplementary Table 3). This observation has clearly suggested that the range-boundary for the kinetic model is functional, and therefore, practically achievable.

Xioameng et al. showed that liposomes with decreasing sizes can be prepared using ultrasonication and by manipulating the buffer to solvent flowrate ratios with a microfluidic device^16^. They have applied ultrasonication in higher range (50–60 kHz) to disintegrate particles. We found that high-frequency ultrasonication is detrimental for mRNA stability (Supplementary Fig. 1c), and therefore, is not suitable for application for delicate alike molecules. On the contrary, we have used lower range of sonication (25 kHz) to provide kinetic energy to the particles to accelerate their movement in the system (Figs. 1b, e, 2a, b). The low-frequency sonication, instead of forced degradation, plausibly have escalated the fusing of LNPs to generate larger LNPs in a controllable manner. This enlargement of the LNPs-size can reasonably be attributed to the enhanced Ostwald ripening phenomenon due to the kinetic force generated by low-moderate sonication.

Primarily, LNP and RNA interact through charge-charge interaction. The interaction is based on the net positive charge on ionizable lipid (e.g., MC3) and the net negative charge on mRNA in a buffer system. The pH of the buffer system may affect overall interaction of lipid and RNA due to the pKa value of the ionizable lipid along with charge-charge interaction. If buffer pH is reduced to acidic range then the interaction with mRNA will be stronger and vice versa; if buffer pH increases to 6.4 (pKa value of MC3), the ionizable lipid may become charge-neutralized where electron association and disassociation is likely in balance, and therefore, MC3 may not interact with RNA at this pH^5,12^. Besides, other factors e.g., osmotic pressure, mechanical condition, thermal properties etc. may impact the charge-charge interaction and thereby can affect mRNA-LNP formation. In this study, the mRNA-LNP formation was achieved at a desired pH and the LNP size was enhanced by application of suitable sonication to harvest LNPs at a desired size-range.

LNPs of different sizes can be prepared by manipulating the process and recipes for LNP formation. The size of the LNP can be manipulated even by tuning the excipient for the formulation. For example, Deng et al. have showed that different sizes of LNPs can be obtained by changing the concentration of an osmolyte, viz., sucrose^17^. A recent study using microfluidic device has shown that lipid concentration and flowrate ratios can significantly affect the particle size and PDI value^12^. They have also showed that the effect of these parameters can be affected by addition of the cargo molecules, viz., siRNA. Different sizes of LNPs over a mean diameter from 50 to 150 nm were produced by adjusting the ratio of the alcohol-to-aqueous volumetric flow rate using microfluidic mixing device^18,19^. Microfluidic device geometry associated with the mixing of aqueous and non-aqueous phases and mass transfer influence the size of LNPs^19^. These methods are suitable for achieving specific size-range of LNPs but not robust enough to produce a desired size-range of LNPs without significant efforts to develop specific method. On the contrary, this is such a robust technology that can provide LNPs within 60 nm to 180 nm size range with a ± 10 nm diameter maintaining the PDI value within the regulatory limit.

LNPs are usually made with an organic solvent necessary for the constituting lipid components. Ethanol has been remained as the most widely used solvent for this purpose. Ethanol must be removed to make the formulation suitable for clinical administration. Two methods are generally adopted in industry to remove ethanol, viz., diafiltration and dialysis. The buffer (generally, at pH 7.4) is exchanged as the media of choice for formulation of the doses during this process. LNPs undergo maturation process during this step as such that smaller LNPs gradually fuse to conform larger LNPs to achieve stabilization^6,20,21^. This phenomenon may not be an exclusive property for LNP made with MC3 but likely a common property for LNPs made with other ionizable lipids as observed for DOTMA-LNPs and DODMA-LNPs^20^. This phenomenon has been collectively attributed to Ostwald ripening, osmotic pressure of the system in action, influence of buffer system on fractional charge of the ionizable lipid, mechanical condition, thermal properties etc. There were efforts to minimize such effects by employing special technologies. The infinite dilution method is a technique in the field for offsetting the effect where, after formation into a suitable buffer, the LNPs are subjected for high dilution to reduce the chance of high-frequency physical contact of LNPs in the system^22^. The final formulation for the method is achieved through buffer exchange or buffer concentration using TFF or diafiltration^21,22^. The other method relies on dialysis against a suitable buffer^6,23^. Both of these methods are in use with appropriate tuning of the method; though the constitutive size enlargement over the time does not stop unless the critical diameter for LNPs for a specific system is achieved^6^. This observation has been suggesting that there might be alternative driving force(s) that can affect Ostwald ripening.

We found that a 2-stage dialysis or solvent exchange, either by dialysis or using TFF, can attenuate the phenomenon (Figs. 1c, 2d, e). Two-stage dialysis has been applied previously by other groups where MES or other buffers were used^24,25^. We have used a new buffer in our system, viz., HEPES-acetate in the first step at pH 6.7 and PBS-based formulation buffer for the second step, that has resulted stable particle size; neither of the two buffers alone could stop gradual size enlargement of the LNPs (Supplementary Fig. 5). DoE analysis has revealed that the HEPES-acetate concentration in buffer treatment is critical for stabilizing the size of nascent LNPs (Fig. 2d, e, Supplementary Fig. 6, 7 and Supplementary Table 4–6). LNPs can be dialyzed against formulation buffer after stabilization without significant variation in sizes of LNPs where buffer strength and pH have critical impacts (Fig. 2f, g, Supplementary Fig. 8, and Supplementary Table 7–9). DoE derived critical buffer compositions stabilized LNPs at 60–180 nm (± 10 nm) diameter range maintaining PDI value ≤ 0.200 for successive processing steps (Supplementary Fig. 9, 10 and Supplementary Table 10, 11).

To obtain LNPs with specific size, while other methods rely on development of a specific process that involves precise composition of components, flowrate ratios, mixing device etc.^4,26^, our method depends on classical DoE model and two simple parameters, viz., pH and sonication time leaving other parameters undisturbed. Maintaining and monitoring of pH of the system and the elapsed time are the easiest critical process parameters compared with any other relevant process parameters for example, chemical compositions, flowrate ratio, mixing mode etc. The tuning of the chemical composition, which requires significant commitments, is not critical in this technique, and therefore, easy to adopt for manufacturing of LNPs in bulk scale for similar formulations. It has been shown that LNPs can continuously fuse together to form larger particle in solution^6,20^ due to the driving force associated with Ostwald ripening^13^. We have exploited this classical phenomenon of the LNP formation and maturation process, and through a systematic sampling approach has determined the suitable condition to obtain desired particle size for LNPs at a given pH (Supplementary Table 12). So that any specific size of LNPs can be obtained within the range of 60 nm to 180 nm for the specific composition of LNP, albeit from lower to higher size with the proceeding of time. The suitable conditions for manufacturing LNPs within the mentioned size is shown in Table 1.

**Table 1:** Critical process parameters for LNP formation, stabilization and formulation.

mRNA-LNPs formation			Stabilization and formulation			
Particles size range (nm)	Required conditions (CPPs)		Required condition (CPPs) for stabilization		Required condition (CPPs) for formulation	
	Buffer pH	Sonication time (s)	Buffer composition	% of buffer	Buffer composition	% of buffer
60–80	~ (4.2–4.7)	20–100	50 mM HEPES, 50 mM sodium acetate, pH 6.7	88 ± 2	1 × PBS, pH 7.2 or 20 mM Tris–HCl, pH 7.2	88 ± 2 100
80–100	~ (4.4–4.7)	10–100				
100–120	~ (4.6–5.0)	10–100				
120–140	~ (4.7–5.0)	25–90				
140–160	~ (4.8–5.0)	35–90				
160–180	~ (4.7–5.0)	70–85				

Every single row in Table 1 reflects a process decision to obtain LNPs within the indicated particle size-range by following relevant values of critical process parameters (CPPs), viz., pH and sonication time. For example, the first row suggests that to obtain LNPs within 60–80 nm diameter the process pH can be maintained within 4.2 to 4.7 and sonication time would be 20 to 100 s. Figure 2a represents a visual description of the situations, where the green band represents the 60–80 nm LNPs. The surface response map reflects that when the pH of the system is 4.2 then 100 s sonication time would produce LNPs with desired size-range, and with the increase in pH value the sonication time would be gradually less. However, the maximum pH value would be 4.7 and the relevant sonication time would be 20 s to obtain LNPs within this size-range. Higher than this pH, e.g., pH 5 would not produce LNPs within this size-range. Accordingly, the system would produce 80–100 nm LNPs at pH 4.5 and 100 s sonication, which cannot be obtained at pH 4.2. At certain condition, the combination of the pH and sonication time is unique. Therefore, though it looks like that values for pH and sonication time in Table 1 are overlapping but practically it is not.

Most of the methods and devices being used for LNP preparation are compromised for preparing sterile LNP preparation for application as injectable drug. Many of these technologies need specific devices, e.g., micro-mixing cartridges (or alike), which are very expensive and have limitations on handling large volume batch preparation. A suitable process engineering design has been reported applicable for manufacturing bulk size batches using ethanol precipitation method^27^. Though this technology is suitable for making large batch but the cleaning (CIP) and sterilization (SIP) process for the relevant system is cumbersome, as well as this technology is not suitable for making LNPs with dynamic size-range. Furthermore, the process vessel of the system needs to be changed based on the batch size that require higher capital investment and bigger footprint. Our system, on the other hand, is based on a simple T-mixer conjugated with in-line coil-flow cell system made with any suitable materials like polymer or glass or ceramics or metals under a low-frequency sonication field (Fig. 1a, b, f). Due to its simple construction these cells can be easily sterilized, packed, stored, transported, and used. These cells are economical, and therefore, can be discarded after a single run, which eliminates the need of cleaning and sterilization after a batch. Scaling up of the batch sizes can be achieved in continuous mode by simply adding of new cells in parallel, which eliminates the need of process tuning in respect of varying batch sizes (Fig. 1h).

**Figure 3:**
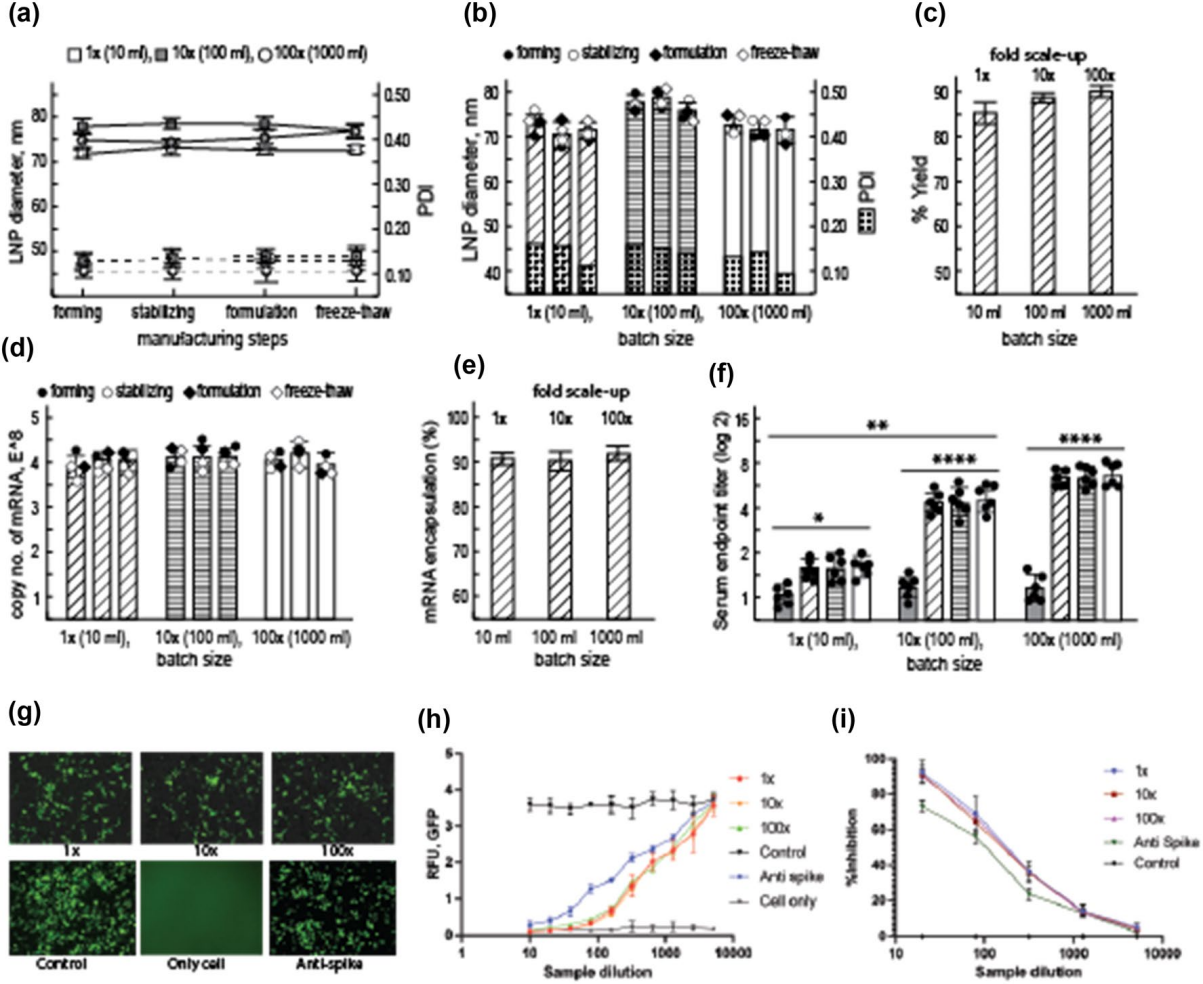
Characterization of the method for scale-up process and evaluation of the bioequivalency of doses obtained from scale-up process. (**a**) The sizes of LNPs do not change during different steps of dose manufacturing and handling; solid line, LNP size and dotted line, relevant PDI values. (**b**) The changes in LNP size for each steps are shown for individual batch for all 3 batch sizes and found non-significant. Relevant PDI values are shown at the bottom in dark shade. (**c**) The yield for different sizes of batches. (**d**,**e**) Copy numbers of mRNA and mRNA encapsulation efficiency in LNP for different size batches, respectively. Data were analyzed using one-way ANOVA and found non-significant; *n* = 4 for all experiments of (**a**–**e**). (**f**) Antibody titer in response of vaccination in rabbits from 3 different size batches (*n* = 6); data were compared by Mann–Whitney test, ****p value < 0.0001, ***p value < 0.001, **p value < 0.01, *p value < 0.05. (**g**) Neutralization of GFP-expressing SARS-CoV-2 pseudovirus in ACE2-expressing HEK cells by vaccinated sera (1 ×, 10 × and 100 × represents vaccines manufactured from different batch sizes). The data shown here is from 640 × dilution of sera. Compare with a commercially available anti-spike antibody all vaccinated sera showed reduced number of GFP-positive cells (*n* = 4). (**h**) The neutralization curve shows the immunized rabbit sera outclassed the commercial SARS-CoV-2 antibody with no significant differences for 3 different size batches (*n* = 4); the IC_50_ values were found at 480 ± 30 × dilutions. (**i**) HIV1-based SARS-CoV-2 pseudovirus neutralization by vaccinated sera was evaluated by RT-PCR (*n* = 4); the IC_50_ values were found at 280 ± 40 × dilutions and superior than the commercial antibody.

The yield percentage and the efficiency of mass balance are two key determinants for the robustness of processes. To check the productivity (process robustness and yield) and functional significance (bioequivalence) of the produced LNPs, we have considered an mRNA-based vaccine candidate against SARS-CoV-2 virus^28,29^. We found that the LNPs within the 60 to 120 nm have produced suitable antibody response in mice model^28,29^. LNPs out of this range elicited weaker response; particularly, larger than 140 nm LNPs did not produce any detectable antibody in mice^28,29^. According to the DoE-derived process, we have produced mRNA-LNPs with particle size 75 ± 10 nm (Supplementary Table 12. The values for relevant productivity parameters were found in close proximities between 3 different scales of batches (Fig. 3a–c, Supplementary Fig. 11–13, and Supplementary Table 12–14) and established the similar process capability in any size of batches. Importantly, all doses were found free of bioburden (data not shown), which is a critical qualification for parenteral dose preparation. To scale-up the process, unlike other processes, addition of the mixing chamber in parallel and maintaining the same flow rate were found fitting. In our study we have scaled up the process successfully from 1 ml/min (manual process) to 10 ml/min (semiautomatic process) to 60 ml/min (continuous process) that can translate into 60 ml/h to 600 ml/h to 3600 ml/h in continuous mode, which has been signifying that the process is robust and easy for adjustment from R&D to manufacturing scale and vice versa.

The doses from the R&D-, pilot- and largescale-processes were tested chemically and found equipotent, which has confirmed dose similarities (Fig. 3d, e). However, it is essential to characterize the bioequivalence of doses produced from different processes to confirm that the doses from all 3 different-size batches are similarly bioactive and can be applied interchangeably. Though the vaccine was previously characterized in mice model^28,29^, rabbit was used in this study to apply larger dose. The mRNA-LNP vaccine did not show any reportable toxicity in animals (Supplementary Fig. 14). Compared to the lower dose (1 µg/50 µl) used in mice, the larger dose (10 µg/500 µl) was favorable to keep the relative volume error lower between doses. The hypothesis was that the equipotent antibody responses shall be obtained from the doses produced from 3 different size batches. The similar level of antibody titers with comparable neutralization capacity against two different SARS-CoV-2-pseudoviruses were highly remarkable that has clearly established the bioequivalence of doses produced from the R&D-, pilot-, and large-scale processes (Fig. 3f–i). Hereby, our study has established a simple and robust process for producing clinical grade LNPs, which can be used for delivering RNAs (and/or other drugs) to achieve desired biological response.

## Conclusion

Collectively, we presented here a novel aspect of size regulation of LNPs using combined effects of pH and sonication. The phenomenon has been exploited to produce LNPs with desired size in continuous mode satisfying regulatory requirement. This report may facilitate relevant research and manufacturing of pharma-grade LNP products. Though we have reported here manufacturing of LNP formulation within 60–180 nm, nevertheless further study can reveal suitable conditions for extended range of LNPs. For example, LNPs around 40 nm has been shown recently is suitable for delivery to the lungs^9^, and extended study of our technology can be helpful for manufacturing such a desired size-range of LNPs. The pharmaceutical science has entered into a new era with the approval of first-in-class mRNA-LNP vaccine for SARS-CoV-2. As per available information, numbers of mRNA-LNP vaccine/drugs are under development and clinical evaluation, and numerous new research programs in this field have been soaring^30–40^. The device is simple, inexpensive, single-use disposable platform and scalable, and therefore, easily adaptable for global application. This simple, robust, continuous process platform can significantly boost the development and manufacturing of mRNA-vaccine/drugs in bulk scale by eliminating technological bottleneck, which may revolutionize the field and ensure quick availability of latest life-saving drugs to the global populations at an affordable cost.

## Materials and methods

### Materials

Cholesterol (Nippon Fine Chemical Co., Ltd, Japan), Dlin-MC3-DMA (AVT Pharmaceutical Tech Co. Ltd., China), DSPC (Nippon Fine Chemical Co., Ltd), DMG-PEG2000 (AVT Pharmaceutical Tech Co. Ltd., China), Ethyl alcohol (Tedia, USA), sodium acetate (FUJIFILM Wako Pure Chemical Corporation, Japan), acetic acid (Sigma Aldrich, Germany), water for injection (WFI), HEPES (FUJIFILM Wako Pure Chemical Corporation, Japan), sodium acetate (FUJIFILM Wako Pure Chemical Corporation, Japan), sodium hydroxide (Merck Germany), disodium hydrogen phosphate (FUJIFILM Wako Pure Chemical Corporation, Japan), potassium dihydrogen phosphate (FUJIFILM Wako Pure Chemical Corporation, Japan), sodium chloride (Merck, Germany), potassium chloride (FUJIFILM Wako Pure Chemical Corporation, Japan) were used in relevant experiments. Water for injection (WFI) was used for water.

Seven Excellence pH/ion meter (Mettler Toledo, Switzerland), Excellence Plus High-Performance Microbalance (Mettler Toledo, Switzerland) and Precision balance (Mettler Toledo, Switzerland), General T-mixture (In-house developed), SB—4200DTS sonication system (Ningbo science Biotechnology Co., Ltd., China) were used. The AKTA flux S and AKTA flux 6 TFF system (GE Healthcare, Sweden) and 0.1 m^2^ NMWCO 100 kDa PES cassette (Sartorius Stedim, Germany) were used in buffer exchange step for 100 × scale-up batch.

### mRNA production

The in vitro transcription (IVT) of mRNA was performed with S-adenosylmethionine and 3′-O-Me-m7G(5′)ppp(5′)G RNA Cap analog (NEB, USA) using MEGAscript™ T7 Transcription Kit (ThermoFisher, USA), and Ribonucleotide Solution Set (NEB, USA); final concentration of ribonucleotides was as follows: ATP and UTP—13.13 mM, and GTP and CTP—9.38 mM. The reaction was carried out for 2 h at 37 °C followed by DNase treatment for 15 min at 37 °C. Dephosphorylation was done using Antarctic Phosphatase (NEB, USA) according to supplier’s manual. IVT capped mRNA was purified using phenol:chloroform:isoamyl alcohol, and MEGAclear™ Transcription Clean-Up Kit (ThermoFisher, USA).

### Stability of mRNA after sonication

Purified mRNA stability was tested after 25 kHz and 50 kHz sonication treatment in water bath. At first, 1 ml purified mRNA was taken in a tube and placed in the sonication field of water sonication bath; thereafter mRNA samples were subjected for either 50 kHz or 25 kHz sonication for 180 s. The mRNA samples (before sonication, after 25 kHz sonication, and after 50 kHz sonication) were analyzed in SEC-HPLC (Ultimate 3000, ThermoFisher, USA).

### Formulation of mRNA-LNPs

All activities were performed in ISO class 7 working area. The mRNA-LNPs formulation process was conducted following the process decision tree and flow diagram (Fig. 1d); detail description is given below.

### mRNA-LNPs formation

The optimum condition of mRNA-LNPs formation was revealed through DoE approach. 6.25 mM sodium acetate buffer at different pH were used to find out the optimum buffer pH condition for mRNA sample preparation. The lipids MC3, DSPC, Cholesterol and DMG—PEG_2000_ were diluted to a working solution at the molar ratio of 50:10:38.5:1.5. After sterile filtration through 0.22-micron nylon filter, lipid mixture and mRNA solution were mixed through T-mixer maintaining a flow rate ratio of 1:3 (flow rate ratio, lipid composition: aqueous = 12.5 ml/min: 37.5 ml/min). Total 54 batches are prepared in 3 different buffer groups, viz., pH 4.18, pH 4.5 and pH 5.0. The samples were subjected for sonication at 25 kHz for indicated time points. The LNP samples were purified by SEC, and filtered through 0.22-micron PES filter for bioburden reduction. The particle size and PDI of LNPs were analyzed as IPC.

### DoE of LNP formation and size optimization

The pH and time dependent (with 3 levels of pH optimality and 0–100 s time-span) randomized 2-level factorial DoE design was obtained using DesignExpert 13 software (Stat-Ease, USA). The model was further potentiated by 5-levels of time constraints at 0, 20, 40, 60, 80, and 100 s with 3 replicate points (blocks). The dataset from the surface response map was analyzed in DesignExpert 13 for the validity of the DoE model. Poisson regression method was followed for the calculation of maximum likelihood analysis where *p* values less than 0.0500 indicate model terms are significant.

### Cavitation zone/sonication field in water bath

The optimum sonication filed was identified for water bath sonication system by aluminum foil signal measurement method^41^. Briefly, aluminum foil was placed in sonication bath at the bottom, then the bath was filled with water up to the desired level and ultrasonication was applied for 2 min. The damaged spots on the foils were marked and a surface response map was plotted. The same process was repeated at 1 inch interval from the bottom to the top of the water level. Relevant damage-spots on the aluminum foils were used and surface response maps were plotted; the 3D zone was identified from surface response maps.

### Stabilization of LNPs

LNP samples were subjected for dialysis for approximately 18 h. Samples were further dialyzed in 100 kDa regenerated cellulose (RC) tube at 22 ± 2 °C against indicated buffers (50 mM HEPES pH 6.7, 50 mM HEPES/sodium acetate pH 6.7, 1 × PBS pH 7.2 and 1 × PBS pH 7.4) at 3 different dilutions (75%, 90%, and 100%) for stabilization of LNPs. Samples were measured at indicated time point for particle size and PDI using Zetasizer Nano ZSP.

### Formulation of LNP

Formulation of LNPs for representative samples were achieved by dialysis using 100 kDa RC tube at 22 ± 2 °C for 3–4 h in indicated buffers following classical screening design; the particle size and PDI were analyzed as IPC. Formulated samples were subjected for dose qualification as described previously^29^.

### Process scale-up

The process was evaluated to confirm whether the process is reproducible and scalable. 1 × (10 ml) and 10 × (100 ml) batches were done manually using a T-mixer and syringe. For 100 × (1000 ml) batch, continuous mode process was used.

#### *Process scale-up at 10* ×

For each 100 ml batch preparation, 30 ml lipid mixture and 90 ml mRNA solution were prepared in relevant buffers (from stock), and passed through sterile 0.22-micron nylon and PES filter, respectively. Lipid mixture and mRNA solution were mixed through T-mixer maintaining flow rate ratio 1:3. The formed LNP were subjected to sonication for indicated time points for size optimization. LNP samples were dialyzed against 88% of 50 mM HEPES/sodium acetate, pH 6.7 for 18 h followed by a second dialysis against 88% of 1 × PBS, pH 7.2. for 3 h. The LNP samples were filtered through 0.22-micron PES filter for bioburden reduction and were subjected for QC analysis as described elsewhere^29^. The particle size and PDI were analyzed as IPC.

#### *Process scale-up at 100* ×

For each 1000 ml batch preparation, 300 ml lipid mixture and 800 ml mRNA solution were prepared in relevant buffers (from stock), and passed through sterile 0.22-micron nylon and PES filter, respectively. Lipid mixture and mRNA solution were mixed through T-mixer maintaining flow rate ratio 1:3 in a continuous mode, and passed through sonication coil (sonicated for indicated time), and collected in 1st TFF reservoir. LNP sample were stabilized in 1st TFF against 88% of 50 mM HEPES/sodium acetate, pH 6.7 through 100 kDa molecular weight cut-off filter. The retentate sample were collected in 2nd TFF reservoir for formulation, where 20 mM Tris–HCl, pH 7.2 was used as formulation buffer. The retentate sample was collected in a sterile pyrogen-free 2 D bag after sterile filtration through Sartopore 2 filter (0.45|0.2-micron PES, Sartorius). Formulated samples were subjected for QC analysis as described elsewhere^29^. The particle size and PDI were analyzed as IPC.

### Lipid nanoparticles (LNPs) size and PDI analysis

Lipid nanoparticle size distribution (size and polydispersity) were analyzed using Malvern Zetasizer Nano ZSP system along with single use, clear, disposable sizing cuvette (DTS0012). For each sample preparation, 975 µl filtered relevant buffer/dispersant was taken in a cuvette (DTS0012) and then mixed well with 25 µl filtered sample by pipetting. The run method was prepared for measurement type—‘size’ in the ‘measure’ tab in the Zetasizer software where cell equilibration temperature (20 °C), equilibration time (300 s), measurement angle (173 °C Backscatter ‘NIBS default’), measurement duration (automatic), delay between measurement (60 s), data processing or analysis model (general purpose ‘normal resolution’) were set. The respective refractive index (RI), viscosity and dielectric constant (DC) were used for analysis. The relevant values are as follows: RI: 1.46, viscosity:1.00 and DC: 72 for 6.25 mM sodium acetate pH 4.18, pH 4.50 and 5.00, RI: 1.464, viscosity:1.00 and DC: 72 for 50 mM HEPES pH 6.7, 50 mM HEPES/ACETATE pH 6.7, 50 mM HEPES pH 7.2, RI: 1.454, viscosity:1.00 and DC: 79 for 1 × PBS pH 7.2 and 7.4 and RI:1.350, viscosity:1.00 and DC: 79 for 20 mM Tris–HCl pH 7.2. After completion of analysis, the size distribution by number percentage and histogram with oversize curve for each sample was deduced.

### Analysis of copy number and percentage of encapsulated mRNA in LNP

RT-qPCR technique was performed according to GoTaq®1-Step RT-qPCR (Promega, USA) kit instructions using LNPs and RNase treated LNP samples. Reverse transcription was done at 37 °C for 15 min then hold for 10 min at 95 °C for reverse transcriptase inactivation and GoTaq® DNA polymerase activation. Primers, 0751F and 592R, were used for amplification of the target. Denaturation was done at 95 °C for 10 s, annealing at 45 °C for 30 s, extension at 68 °C for 30 s for 40 cycles (QuantStudio 12 K Flex, ThermoFisher, USA). After completion of PCR cycle, melt curve was analyzed for integrity checking.

### Animal management and vaccination

The study is reported in accordance with ARRIVE guidelines. A total of 24 New Zealand white rabbit (female) of 10–12 weeks old were selected and isolated 5 days before immunization. After careful observation and conditioning, they were subjected for vaccination. The temperature in the experimental animal room was 26 °C (± 2 °C) and the relative humidity was 60 ± 5%. The room was HVAC controlled ISO class 7 room with 70% fresh air intake and full exhaust. The rabbits were individually housed in cage with proper water and food, and kept under 12 h of day-night cycle. Animal cages were cleaned and locations of cages were rotated daily. Animals were separated into 4 different groups consisting 6 rabbits in each group. Randomization was done using the standard = RAND() function in Microsoft Excel; treatments were given single-blinded in numerical order. GBPD060 formulations (from 1 ×, 10 ×, and 100 × batch size) and vehicle were administered intramuscularly (IM) in quadriceps as per study design for treatments and placebo, respectively. The study plan and procedures were approved by the internal ethical review board (IECB-PCS: Internal Ethical Clearance Board for Pre-Clinical Study) of Globe Biotech Limited, which is complied with the local and international regulation.

### Antibody titer analysis

Serum from the rabbit of different groups were analyzed by enzyme‐linked immunosorbent assay (ELISA) to determine sera antibody titers. ELISA plate (Corning, USA) was coated with 1 µg/ml SARS-CoV-2 Spike S1 + S2 ECD-His recombinant protein (Sino Biological, China) in Dulbecco’s phosphate-buffered saline (DPBS) (ThermoFisher, USA) for 2 h at room temperature. Plate was washed for 3 times with DPBS + 0.05% Tween 20 (Scharlau, Spain) and then blocked with PBS + 1% BSA (ThermoFisher, USA) + 0.050% Tween-20 for 2 h at 37 °C. The plate was washed for 3 times then incubated with rabbit sera and SARS-CoV-2 Spike antibody (Sino Biological, China) for 2 h at 37 °C. After washing for 3 times, the plate was incubated with HRP-conjugated goat anti-rabbit IgG (H + L) secondary antibody (ThermoFisher, USA) for 50 min at room temperature. Final washing was done for 3 times and then developed for colorimetric reaction with Pierce TMB substrate (ThermoFisher, USA) for 10 min. The reaction was stopped with 1 N HCl and the plate was read at 450 nm wavelength within 30 min.

### Pseudovirus preparation and in vitro neutralization

Adenovirus and retrovirus based pseudovirus were prepared for SARS-CoV-2 in vitro and in vivo neutralization assay as described previously^28,29^.

### Neutralization assay

ACE2-expressing HEK293 cell (Innoprot, Spain) were seeded in two 96-well TC-treated plate at a concentration of 2.2 × 10^4^ cells/well and incubated for overnight. One plate was used for adeno-based pseudovirus and other plate for retro-based pseudovirus, respectively. Two separate plates were used for serum preparation. Different rows of the plate were used for different group, such as A1–A10 for treatment group 1 (1 ×: 10 ml batch), B1–B10 for treatment group 2 (10 × : 100 ml batch), C1–C10 for treatment group 3 (100 ×: 1000 ml batch), D1–D10, E1–E10 and F1–F10 for control, commercial anti spike and only cell group, respectively. Sera from different mice of same group were collected and pooled for neutralization assay. 10 µl sera from vaccinated mice was added in 90 µl complete DMEM media, and were serially twofold diluted up-to 9 times. 1.2 × 10^5^ pseudovirus in 50 µl was added into different wells that contained serially diluted serum and mixed properly. The SARS-CoV-2 pseudovirus and serum mixture was incubated for 1.5 h at 37 °C. Then, 100 µl of pseudovirus and serum mixture was transferred on pre seeded cells. 5 µg/ml poly L-lysine (Wako, Japan) was added into each well for enhancing the transduction. Then, incubation was performed at 37 °C for 48 h and GFP-fluorescence were measured using Varioskan LUX (ThermoFisher, USA). Number of virus particle inside the cells were determined by qPCR (QuantStudio 12 K Flex, ThermoFisher, USA).

### Statistical analysis

DesignExpert 13 and Microsoft Excel were used for statistical analysis. The standard deviation was considered as data acceptance criteria; *p* < 0.05 were considered significant.

## Supplementary Information

Supplementary Information.

## Data Availability

The data that support the findings of this study are available within the article and its supplementary document file, or are available from the corresponding author upon reasonable request.
