# Supplementary material for: DoE-derived continuous and robust process for manufacturing of pharmaceutical-grade wide-range LNPs for RNA-vaccine/drug delivery
Source: Sci Rep. 2022 Jun 7;12:9394. doi: 10.1038/s41598-022-12100-z (PMC9172984; doi:10.1038/s41598-022-12100-z)
Supplement: Supplementary file 1 — Supplementary Information. [file 41598_2022_12100_MOESM1_ESM.pdf]

Appendix A-Supplementary information

a

ID	Run no.	Space type	Factor 1 A: pH	Factor 2 B: time (sec)	Block	Batch no.
2	1	Factorial	4.18	40	3	7-9
11	2	Factorial	4.5	100	3	34-36
9	3	Factorial	4.5	60	3	28-30
12	4	Factorial	5.0	0	3	37-39
15	5	Factorial	5.0	60	3	46-48
4	6	Factorial	4.18	80	3	13-15
3	7	Factorial	4.18	60	3	10-12
6	8	Factorial	4.5	0	3	19-21
17	9	Factorial	5.0	100	3	52-54
8	10	Factorial	4.5	40	3	25-27
14	11	Factorial	5.0	40	3	43-45
0	12	Factorial	4.18	0	3	1-3
5	13	Factorial	4.18	100	3	16-18
10	14	Factorial	4.5	80	3	31-33
13	15	Factorial	5.0	20	3	40-42
1	16	Factorial	4.18	20	3	4-6
16	17	Factorial	5.0	80	3	49-51
7	18	Factorial	4.5	20	3	22-24

b

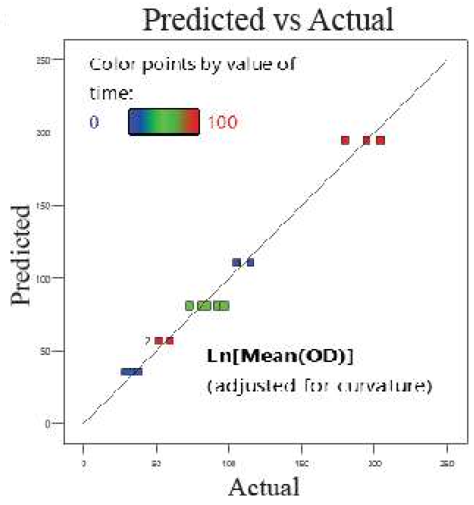

c

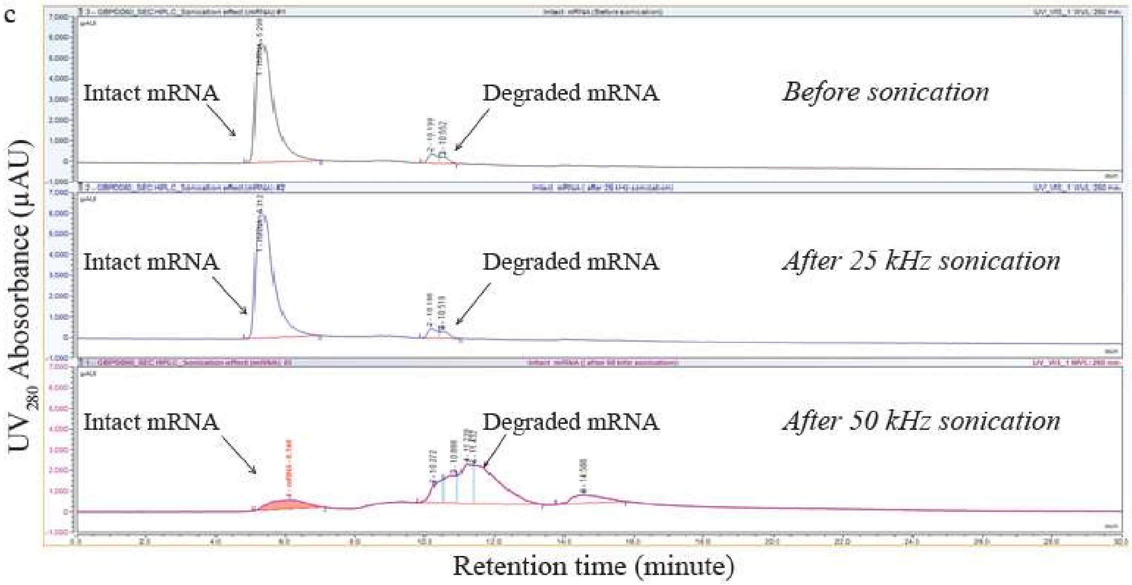

Supplementary Figure 1. The full-factorial DoE plan. (a) DesignExpert V.13 generated DoE plan was potentiated with adjustment of constraints to accommodate desired time intervals in experiments. Total 54 batches were used for the study; the DoE condition IDs are shown with relevant batch numbers. (b) DoE model validation plot represents that the value of predicted ODs for different conditions are aligned with the actual OD values. (Sonication time dependent response, $p<0.001$). (c) High-frequency sonication degrades mRNA. Samples were sonicated at indicated power for 100 sec and analyzed in SEC; higher frequency degraded the mRNA.

Appendix A-Supplementary information

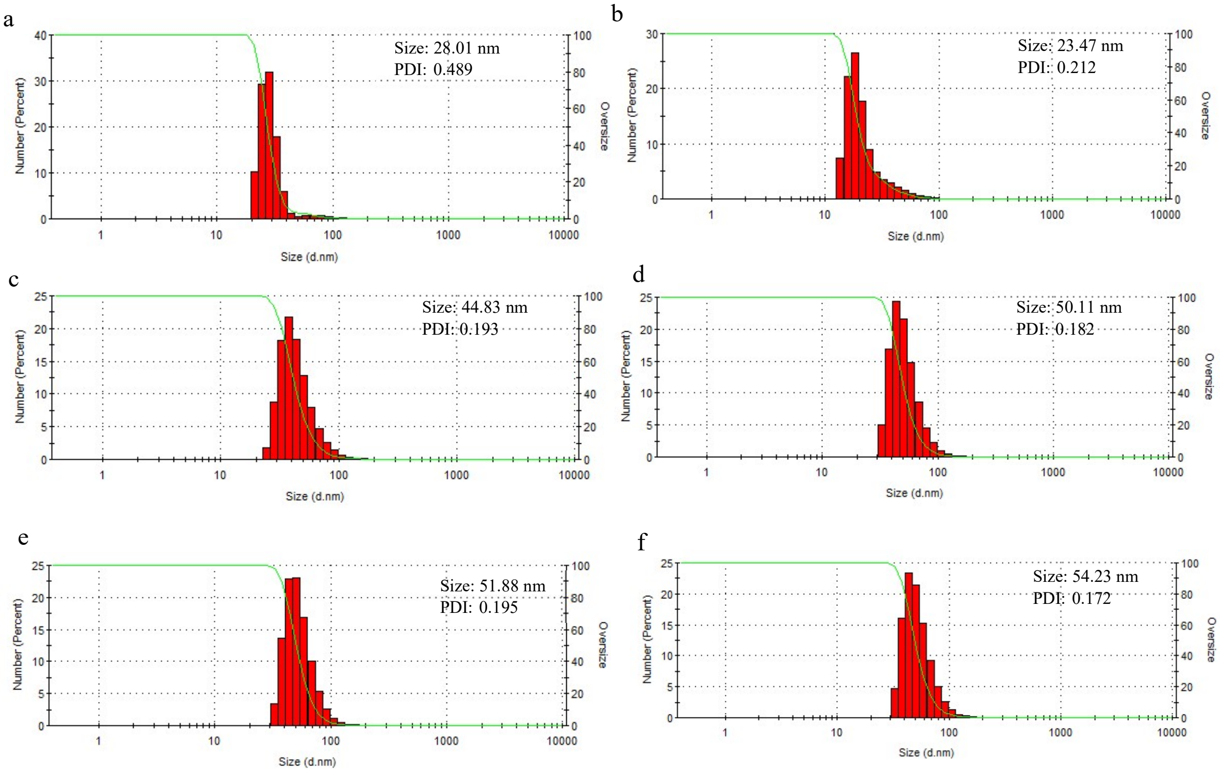

Supplementary Figure 2. Formation of LNP. A representative data set from triplicate experiments is shown. Histogram a, b, c, d, e and f shows the representative sample (*n*=3) of LNPs size distribution after mixing the lipid composition with mRNA solution (prepared with 6.25mM sodium acetate, pH 4.18) by T-mixture followed by sonication (25 kHz) for 0, 20, 40, 60, 80 and 100 seconds, respectively.

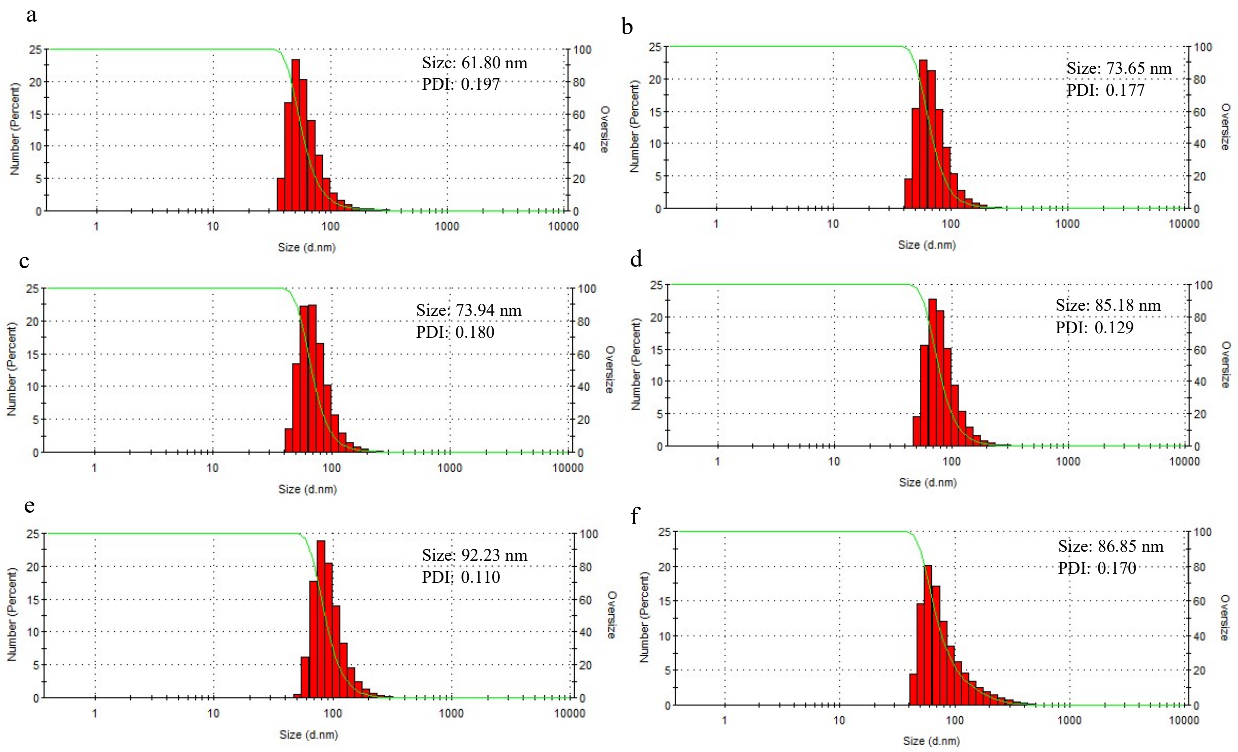

Supplementary Figure 3. Formation of LNP. A representative data set from triplicate experiments is shown. Histogram a, b, c, d, e and f show the representative sample (*n*=3) of LNPs size with PDI value after mixing the lipid composition with mRNA solution (prepared with 6.25mM sodium acetate, pH 4.50) by T-mixture followed by sonication (25 kHz) for 0, 20, 40, 60, 80 and 100 seconds, respectively.

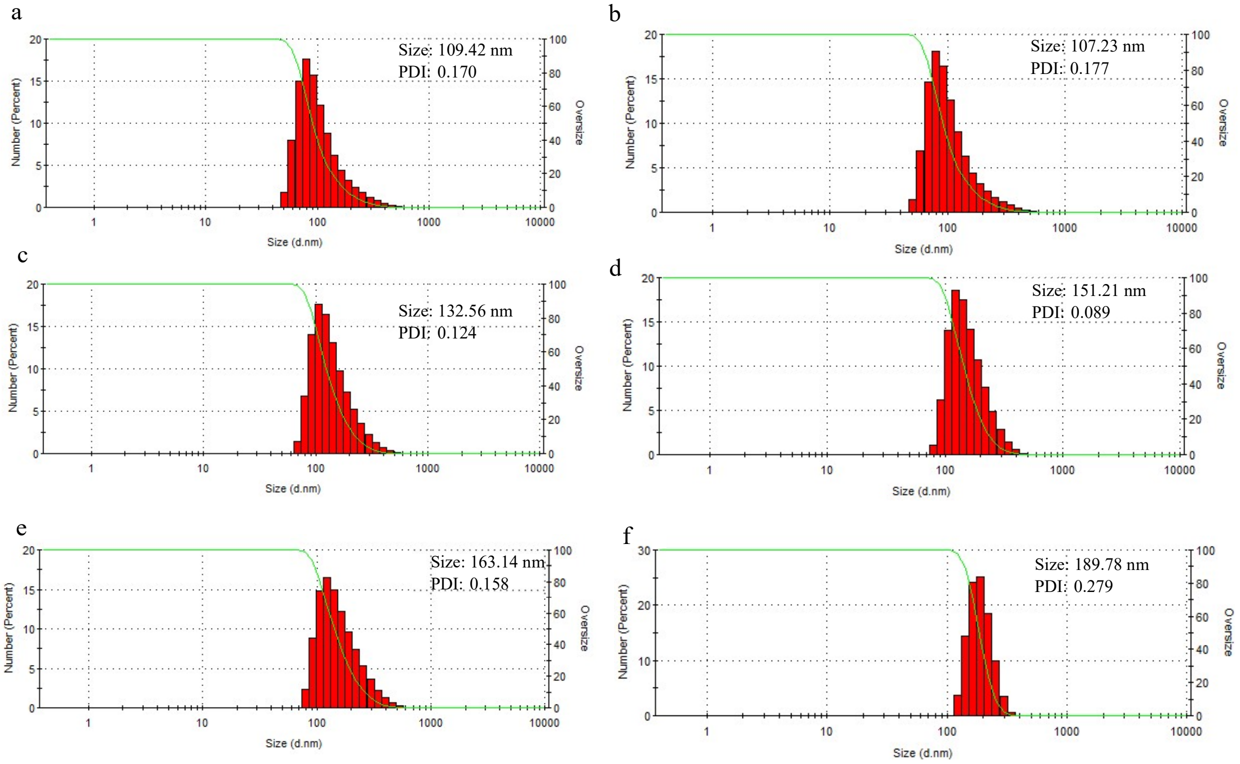

Supplementary Figure 4. Formation of LNP. A representative data set from triplicate experiments is shown. Histogram a, b, c, d, e and f show the representative sample (*n*=3) of LNPs size with PDI value after mixing the lipid composition with mRNA solution (prepared with 6.25 mM sodium acetate, pH 5.00) by T-mixture followed by sonication (25 kHz) for 0, 20, 40, 60, 80 and 100 seconds, respectively.

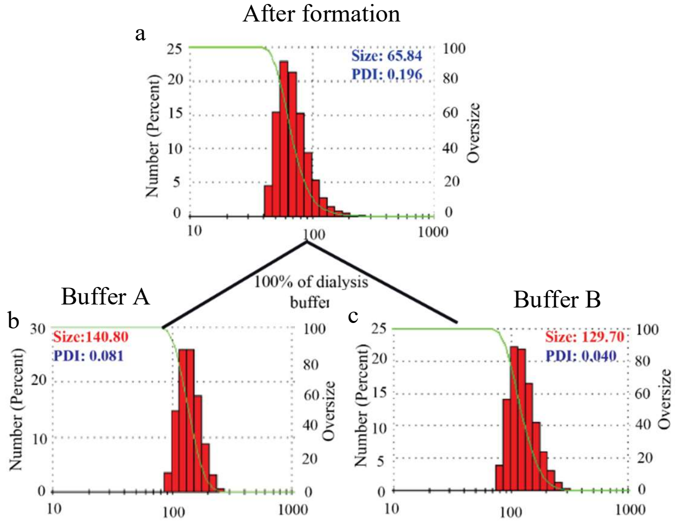

Supplementary Figure 5. Stabilization of representative LNP sample (65.84 nm) from size range 60-80 nm in buffer A (50mM HEPES, pH 7.4) and buffer B (1×PBS, pH 7.4). **a,** represents the accepted particle size after formation. **b**, represents unstable sizes (140.80 nm) after dialyzed against 50mM HEPES, pH 7.4 although the homogeneity remains within acceptance range. **c**, represents unstable sizes (129.70 nm) after dialyzed against 1×PBS, pH 7.4

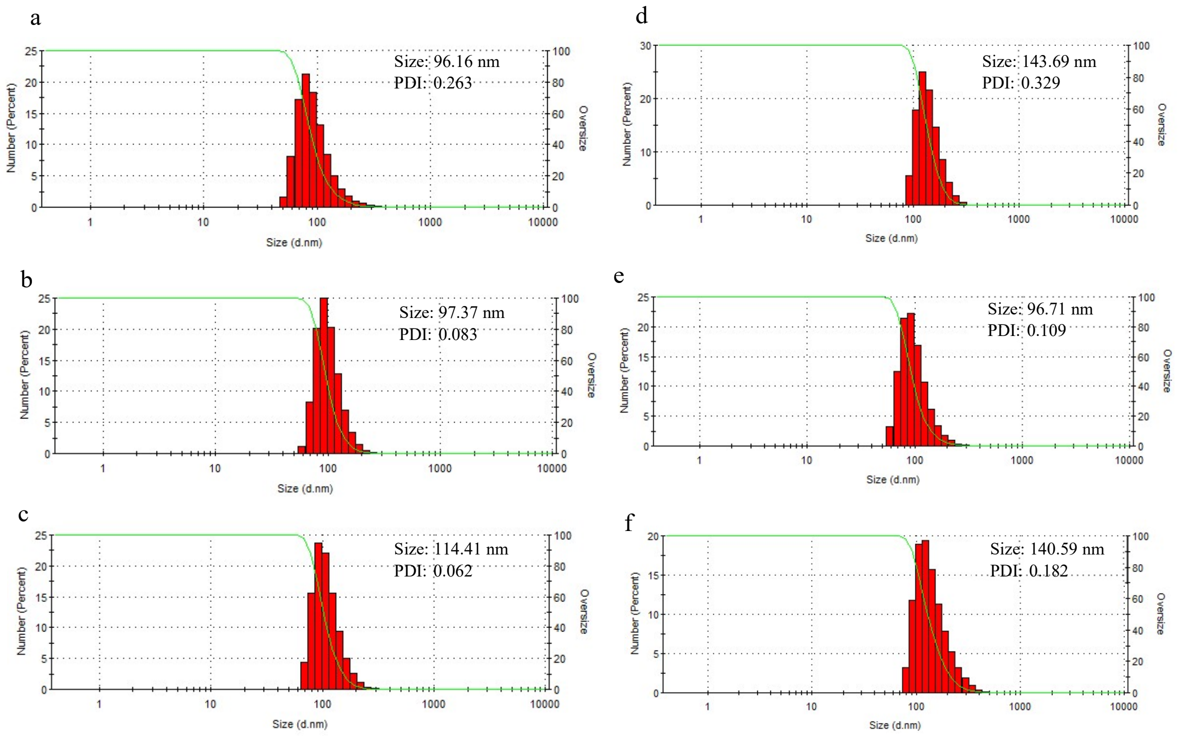

Supplementary Figure 6. LNP stabilization. A representative data set from triplicate experiments is shown. Histograms show the representative sample (*n*=3) of LNPs size with PDI value after dialysis against 75%, 90% and 100% of 1× PBS, pH 7.2 (a, b and c, respectively) and 1× PBS, pH 7.4 (d, e and f, respectively).

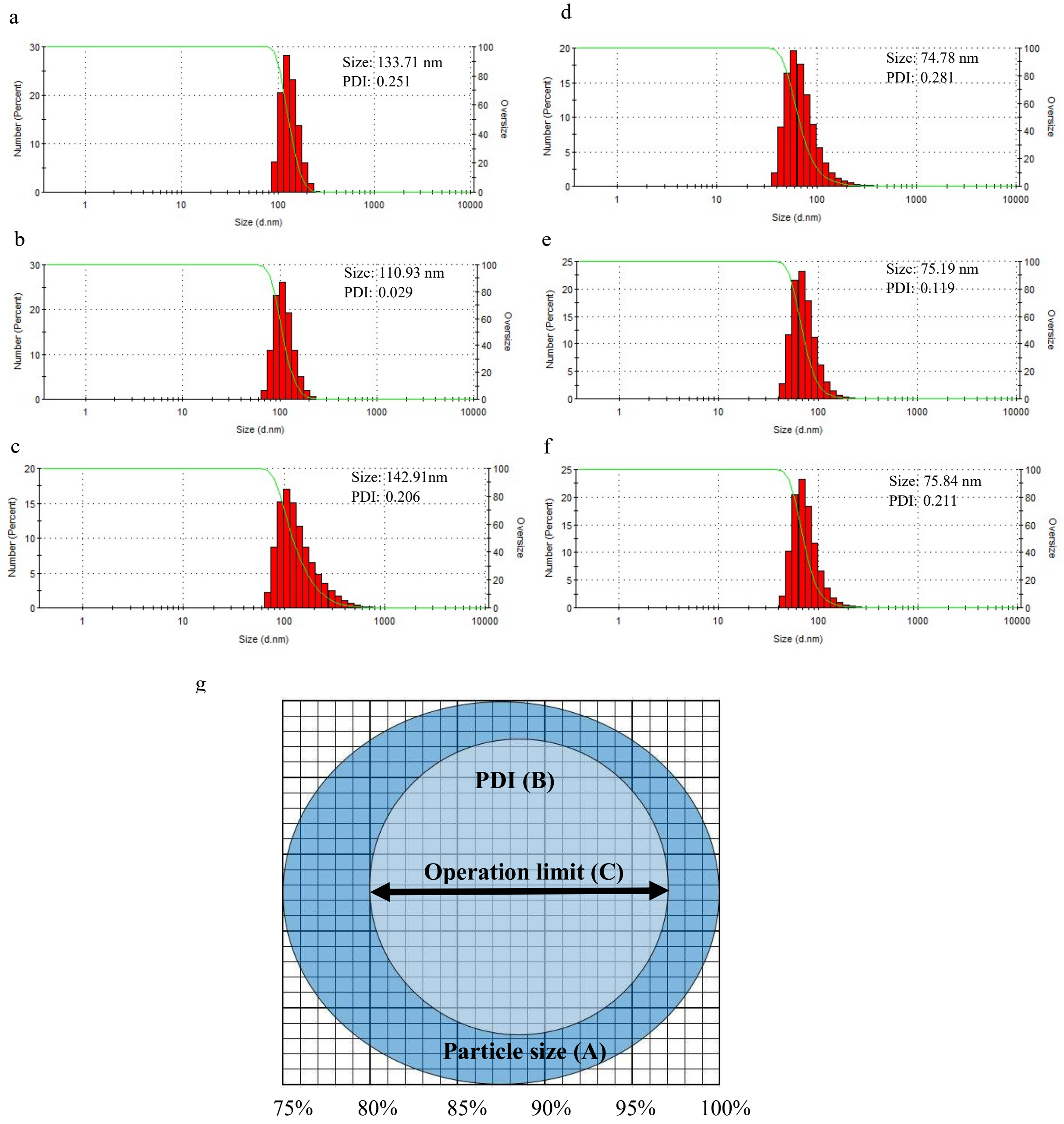

[1 square unit equivalent to 1%]
A: % of respective buffer to obtain accepted particle size range (60-80 nm)
B: % of respective buffer to obtain accepted PDI range (0.000-0.200)
C: % of respective buffer (80-97%) buffer, more preferably 88±2%, to obtain desired particles size distribution (indicated arrow sign)

Supplementary Figure 7. LNP stabilization. A representative data set from triplicate experiments is shown. Histograms shows the representative sample (*n*=3) of LNPs size with PDI value after dialysis against 75%, 90% and 100% of 50 mM HEPES, pH 6.7 (a, b and c, respectively) and 50 mM HEPES/50 mM sodium acetate, pH 6.7 (d, e and f, respectively) for stabilization of particles. **g,** shows the optimum % of dialysis buffer to stabilize the particle size (60 – 80 nm) with PDI (≤ 0.200).

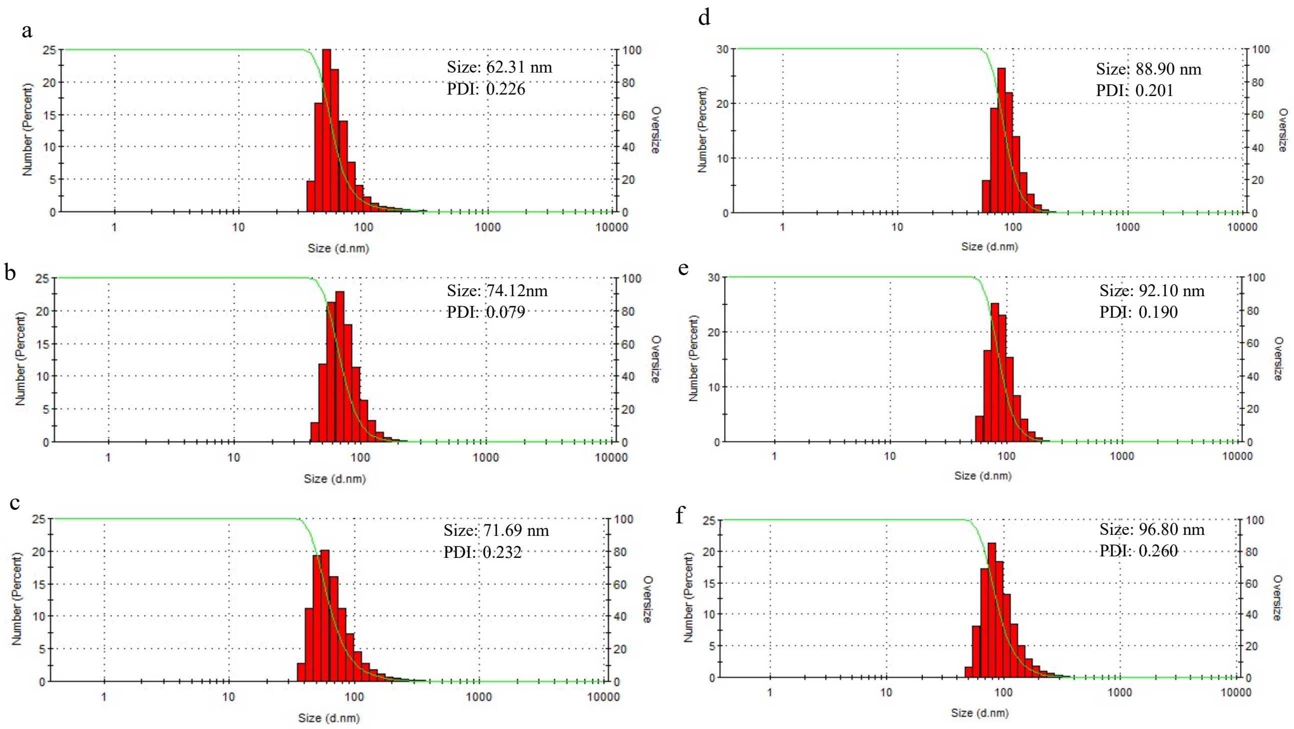

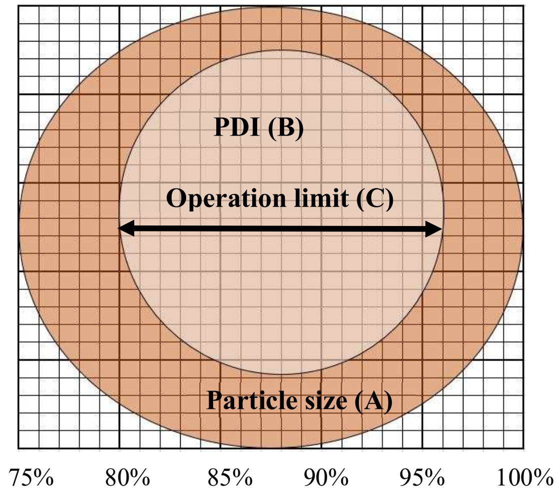

[1 square unit equivalent to 1%]

A: % of respective buffer to obtain accepted particle size range (60-80 nm)
B: % of respective buffer to obtain accepted PDI range (0.000-0.200)
C: % of respective buffer (80-96%) buffer, more preferably 88±2%, to obtain desired particles size distribution (indicated arrow sign)

Supplementary Figure 8. Formulation of stabilized particles. A representative data set from triplicate experiments is shown. Histograms show the representative sample (*n*=3) of LNPs size with PDI value after dialysis against 75%, 90% and 100% of 1× PBS, pH 7.2 (a, b and c) and 1× PBS, pH 7.4 (d, e and f) to formulate stabilized particles. **g**, shows the optimum % of dialysis buffer to formulate the particle size (60 – 80 nm) with PDI (≤ 0.200).

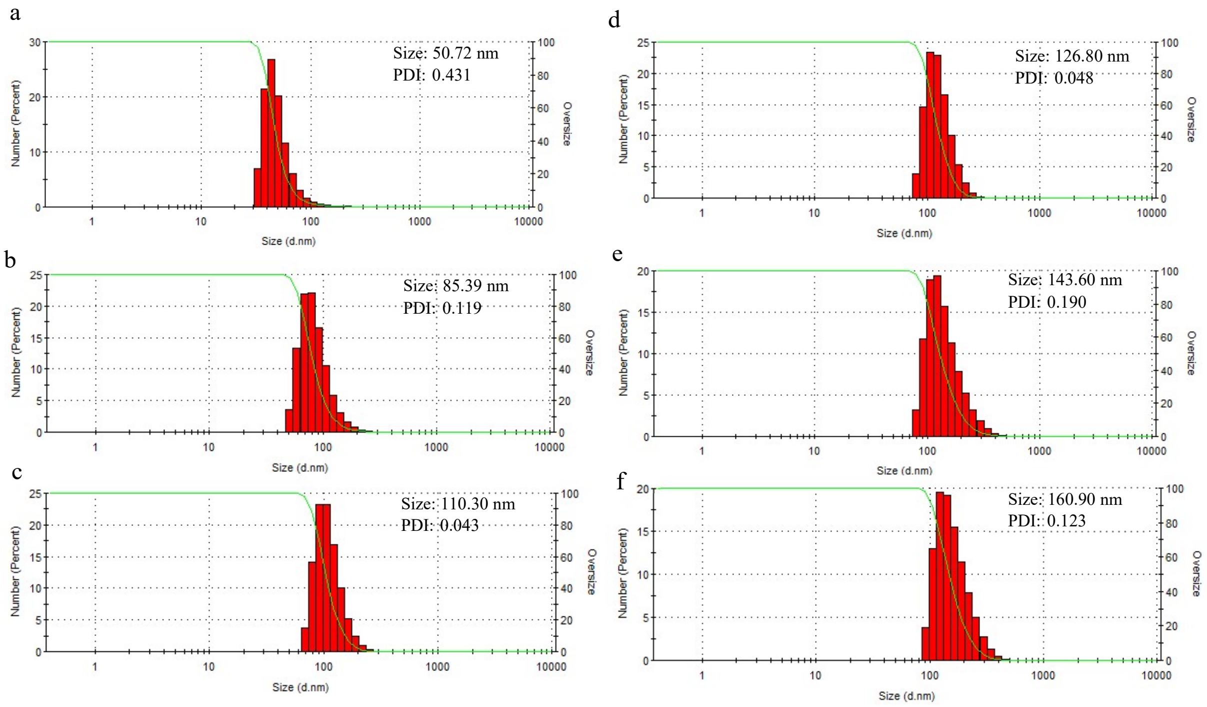

Supplementary Figure 9. Stabilization of different size ranges in 88±2% of 50 mM HEPES/sodium acetate, pH 6.7. A representative data set from triplicate experiments is shown. Histogram a (40-60nm), b (80-100 nm), c (100-120 nm), d (120-140 nm), e (140-160 nm), and f (160-180 nm) show the representative sample (*n*=3) of LNPs size with PDI (≤ 0.200) after dialysis.

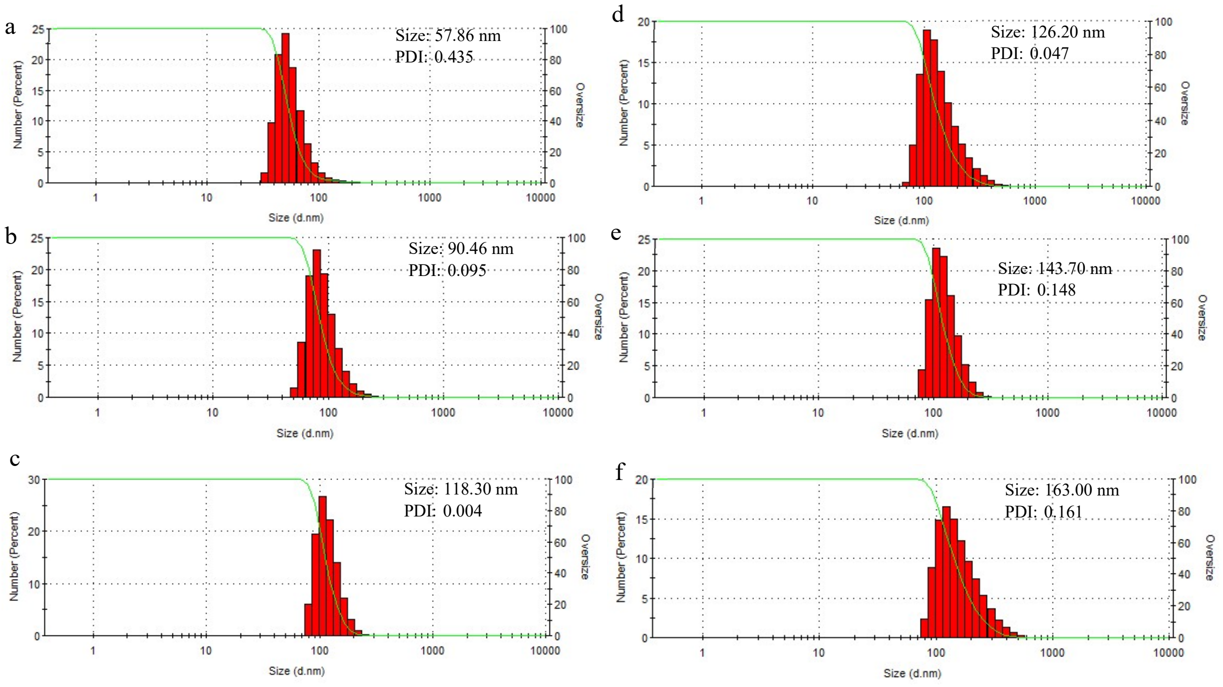

Supplementary Figure 10. Formulation of different size ranges of stabilized particles in 88±2% of 1× PBS, pH 7.2. A representative data set from triplicate experiments is shown. Histogram a (40-60nm), b (80-100 nm), c (100-120 nm), d (120-140 nm), e (140-160 nm), and f (160-180 nm) shows the representative sample (*n*=3) of LNPs size with PDI value (≤ 0.200) after dialysis.

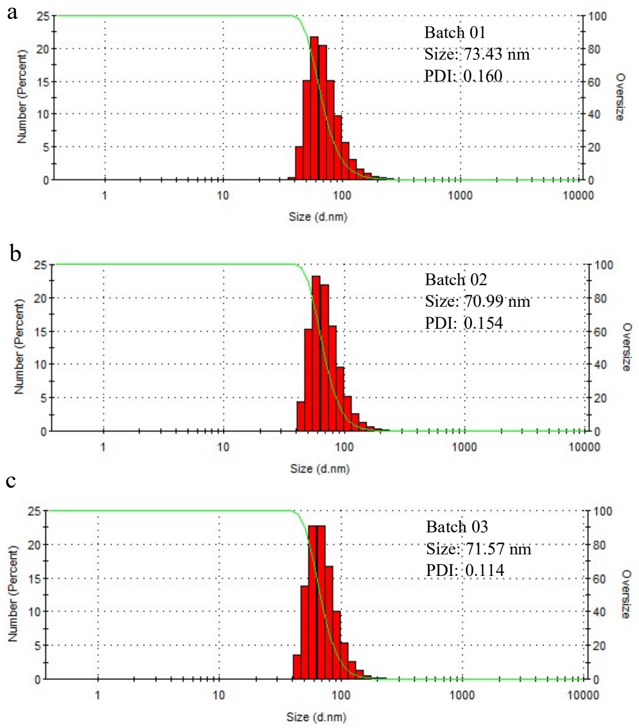

Supplementary Figure 11. Reproducibility of LNP formulation. A representative data set from triplicate experiments is shown. Histogram a, b, and c show the representative sample (*n*=3) of LNPs size and PDI values of three consecutive batch for 1× (10 ml) where sample was formulated in 1× PBS, pH 7.2.

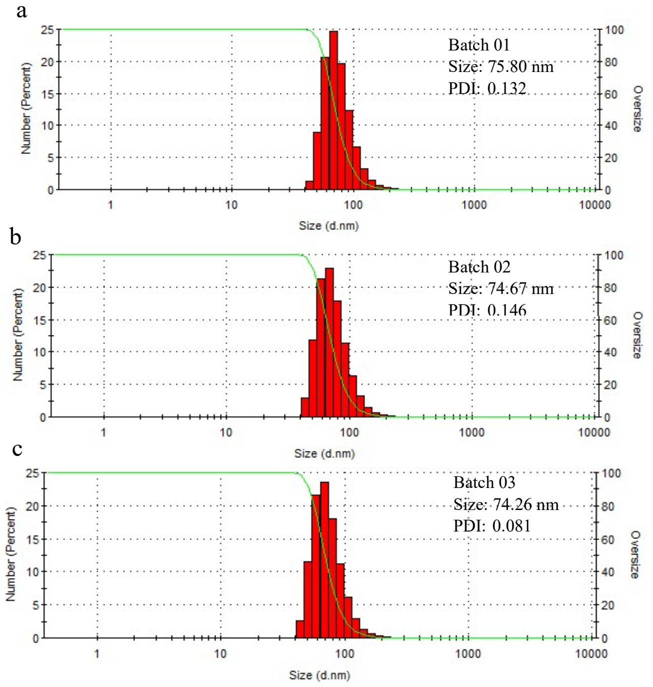

Supplementary Figure 12. Reproducibility and scalability of LNP formulation. A representative data set from triplicate experiments is shown. Histogram a, b, and c show the representative sample (n=3) of LNPs size and PDI values of three consecutive batch for 10× (100 ml) where stabilized particles were formulated in 20 mM Tris.HCl, pH 7.2.

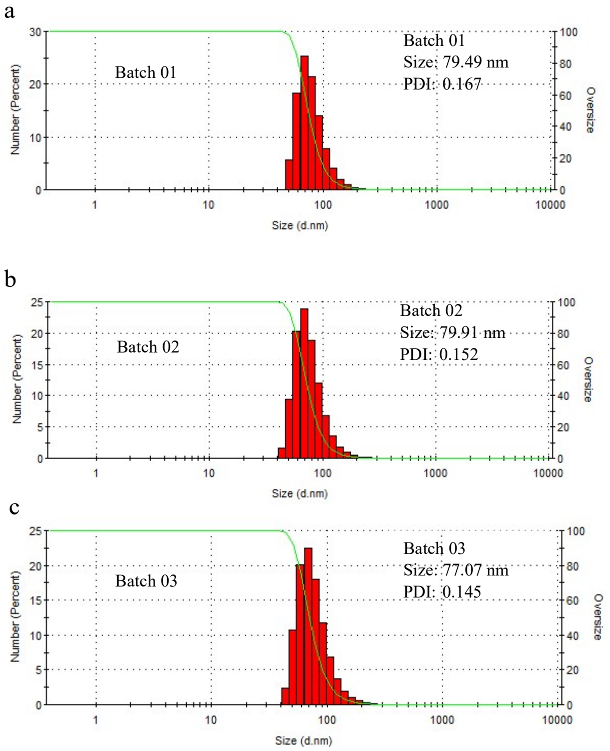

Supplementary Figure 13. Reproducibility and scalability of LNP formulation. A representative data set from triplicate experiments is shown. Histogram a, b, and c show the representative sample (*n*=3) of LNPs size and PDI values of three consecutive batch for 100× (1000 ml) where stabilized particles were formulated in 20 mM Tris.HCl, pH 7.2.

Appendix A-Supplementary information

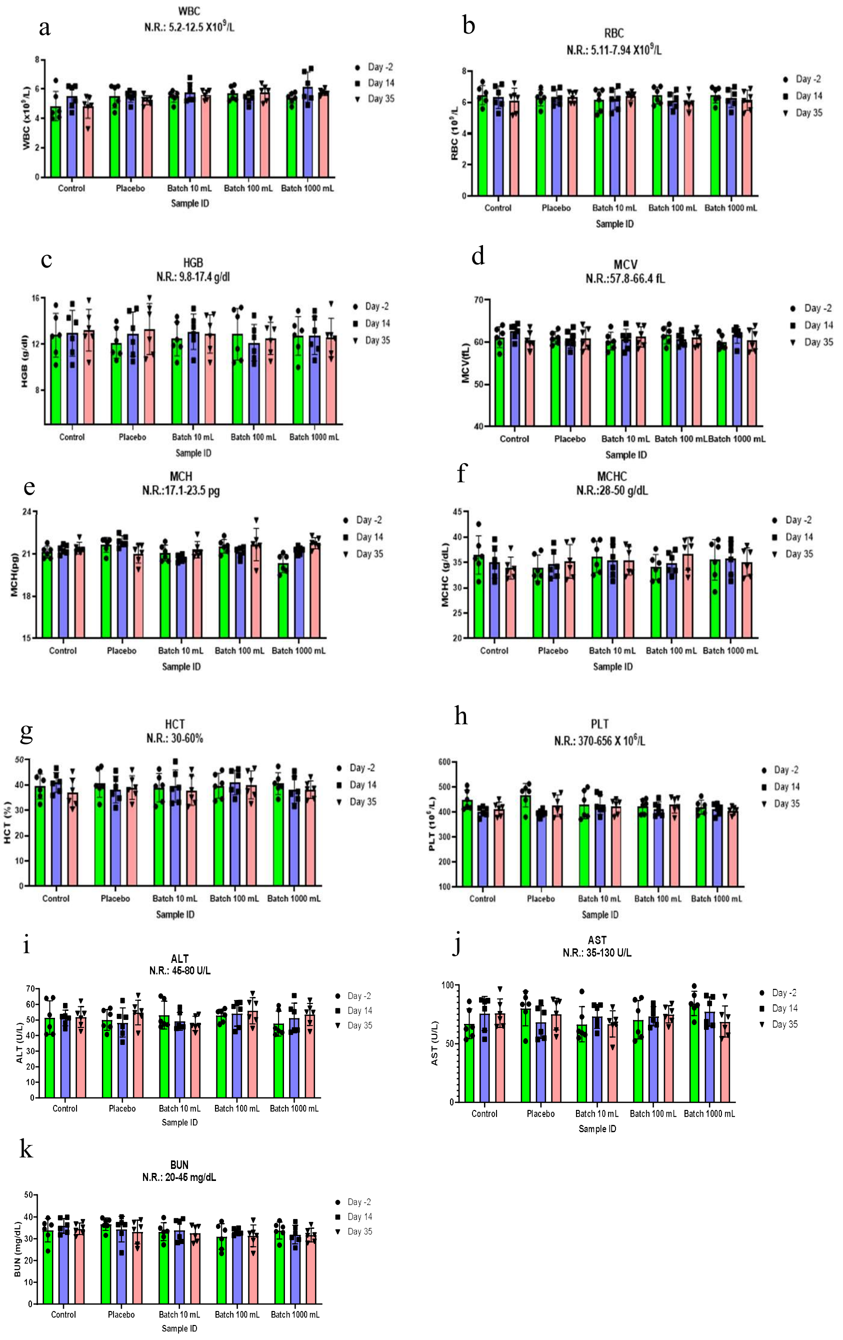

Supplementary Figure 14. Hematology and biochemistry analysis. Graph a represents white blood count (WBC), b represents red blood cell (RBC), c represents HGB, hemoglobin, d represents MCV, mean corpuscular volume, e represents MCH, mean corpuscular hemoglobin, f represents MCHC, mean corpuscular hemoglobin concentration, g represents HCT, hematocrit, h represents PLT, platelet, i represents alanine transaminase (ALT), j represents aspartate aminotransferase (AST), k represents blood urea nitrogen (BUN). Data were analyzed using one-way ANNOVA method, and found statistically non-significant (n=6).

Appendix A-Supplementary information

Supplementary Table 1. Particle sizes with PDI values at different buffer and sonication time (3 replicates)

Sonication time (seconds)	Acetate buffer, pH 4.18		Acetate buffer, pH 4.50		Acetate buffer, pH 5.00	
	LNPs sizes	PDI values	LNPs Sizes	PDI values	LNPs sizes	PDI values
0	28.04±1.02	0.449±0.039	63.82±2.02	0.198±0.003	109.70±1.05	0.168±0.003
20	22.14±1.23	0.241±0.035	72.73±2.57	0.175±0.002	110.40±4.56	0.174±0.004
40	44.85±3.06	0.196±0.003	73.94±2.11	0.180±0.003	130.20±2.15	0.121±0.003
60	52.13±2.02	0.189±0.006	84.07±1.13	0.126±0.003	151.69±1.74	0.094±0.005
80	53.93±2.04	0.195±0.002	93.49±2.67	0.118±0.007	163.00±1.67	0.161±0.003
100	53.04±2.50	0.180±0.007	87.17±0.82	0.169±0.003	190.90±3.53	0.277±0.006

Supplementary Table 2. Interpretation of surface response plot from Fig. 2a, b for mRNA-LNPs formation

Color zone	Particle size range (nm)	Conditions (CPPs)	
		Sonication time (seconds)	Acetate buffer pH ranges
Dark blue	20 – 40	0 – 20	~ 4.2
Dark orange	40 – 60	40 – 100	~ 4.2 – 4.4
Green	60 – 80	20 – 100	~ 4.2 – 4.7
Purple	80 – 100	10 – 100	~ 4.4 – 4.7
Yellow	100 – 120	10 – 100	~ 4.6 – 5.0
Dark blue	120 – 140	25 – 90	~ 4.7 – 5.0
Light orange	140 – 160	35 – 90	~ 4.8 – 5.0
Black	160 – 180	70 – 85	~ 4.7 – 5.0

N.B.: PDI value ≤ 0.200 except size range (20-40 nm).

Supplementary Table 3. Cross-point validation conditions of DoE plot and DLS data respect to mRNA-LNPs sizes and PDI values (3 replicates).

Designed process parameters				Applied process parameters		Result from DLS		Decision
Sl. No.	Expected size (nm)	Buffer pH	Sonication time (seconds)	Buffer pH	Sonication time (seconds)	Particle size (nm)	PDI	Accepted/ Rejected
01	20-40	4.20	10	4.19±0.01	10.33±0.58	30.16±1.75	0.878±0.051	Accepted*
02	40-60	4.30	42	4.31±0.01	42.00±1.00	58.21±2.13	0.180±0.005	Accepted
03	60-80	4.60	11	4.62±0.02	11.00±1.00	73.08±2.52	0.123±0.005	Accepted
04	80-100	4.60	90	4.41±0.02	91.00±1.00	84.76±2.77	0.183±0.004	Accepted
05	100-120	4.80	36	4.82±0.02	35.00±1.00	109.80±2.70	0.143±0.028	Accepted
06	140-160	5.00	94	5.03±0.05	93.00±1.00	153.90±2.59	0.118±0.007	Accepted
07	80-100	4.60	50	4.60±0.02	51.00±1.00	85.66±1.28	0.142±0.037	Accepted

*, Particle size matched but PDI value failed.

Appendix A-Supplementary information

Supplementary Table 4. mRNA-LNPs sizes with PDI values after stabilization at different percentage of buffers (3 replicates)

% of Buffer	50 mM HEPES, pH 6.7		50 mM HEPES/50 mM sodium acetate, pH 6.7		1×PBS, pH 7.2		1×PBS, pH 7.4	
	LNPs sizes	PDI values	LNPs Sizes	PDI values	LNPs sizes	PDI values	LNPs sizes	PDI values
75.0	133.50± 3.17	0.322± 0.063	73.69± 3.11	0.287± 0.066	93.51± 4.00	0.249± 0.031	141.30± 8.81	0.333± 0.017
90.0	111.10± 3.53	0.030± 0.012	74.14± 2.68	0.122± 0.004	101.10± 5.42	0.051± 0.028	100.00± 11.33	0.112± 0.008
100.0	145.80± 2.92	0.244± 0.052	76.70± 2.35	0.221± 0.015	109.40± 5.62	0.076± 0.012	143.60± 7.06	0.190± 0.012

N.B.: Starting samples (pooled; size 72.73±2.57 nm and 73.94±2.11 nm).

Supplementary Table 5. Interpretation of desired size range from surface response plot from Fig. 2d for stabilization

Color zone	Expected particle size (nm)	% of buffer	Exchange buffer	Acceptance criteria (Size should be within 60 – 80 nm)
Green	60 – 80	75 – 100	50 mM HEPES/sodium acetate, pH 6.7	Accepted

Supplementary Table 6. Interpretation of desired PDI from surface response plot from Fig. 2e for stabilization

Color zone	Expected PDI value	% of buffer	Exchange buffer	Accepted criteria (PDI should be $\leq$ 0.200)
Green	0.0 – 0.2	80.0 – 97.0	50 mM HEPES/sodium acetate, pH 6.7	Accepted

Supplementary Table 7. mRNA-LNPs sizes with PDI values after formulation at different percentage of buffers

% of Buffer	1×PBS, pH 7.2		1×PBS, pH 7.4	
	LNPs sizes	PDI values	LNPs sizes	PDI values
75.0	62.86±1.46	0.225±0.004	89.29±0.90	0.205±0.004
90.0	74.26±1.48	0.081±0.002	91.86±2.10	0.160±0.026
100.0	70.92±1.45	0.233±0.005	95.64±1.02	0.240±0.026

Supplementary Table 8. Interpretation of desired size range from Fig. 2f for formulation by buffer exchange

Color zone	Expected particle size range (nm)	% of buffer	Exchange buffer	Accepted criteria (Size should be stable)
Green	60 – 80	75 − 100	1× PBS, pH 7.2	stable

Supplementary Table 9. Interpretation of desired PDI from Fig. 2g for formulation by buffer exchange

Color zone	Expected PDI value	% of buffer	Exchange buffer	Accepted criteria (Size should be stable)
Green	0.0 – 0.2	80.0 – 96.0	1× PBS, pH 7.2	stable

Supplementary Table 10. | Stabilization of different sizes ranges (40 – 180 nm) in 88±2% of 50 mM HEPES/sodium acetate, pH 6.7.

Particle size range (nm)	mRNA- LNPs sizes (nm) after formation	PDI values after formation	mRNA- LNPs sizes (nm) after stabilization	PDI values after stabilization	Acceptance criteria (particle should be stable)
40 – 60	52.13 ±2.02	0.189±0.006	50.73±2.55	0.431±0.003	stable*
80 – 100	84.07±1.13	0.126±0.003	86.31±1.76	0.151±0.001	stable
100 – 120	109.70±4.56	0.168±0.004	112.47±2.23	0.044±0.004	stable
120 – 140	130.20±2.15	0.121±0.003	125.85±0.92	0.050±0.007	stable
140 – 160	158.60±1.74	0.094±0.005	144.87±1.61	0.189±0.005	stable
160 – 180	163.00±1.67	0.161±0.003	161.13±1.41	0.124±0.004	stable

*, Particle size matched but PDI value failed and % of buffer achieved by TFF process

Supplementary Table 11. Formulation of different sizes ranges (40 – 180 nm) of stabilized particles in 88±2% of 1× PBS, pH 7.2.

Particle size range (nm)	mRNA- LNPs sizes (nm) after stabilization	mRNA- LNPs PDI values after stabilization	mRNA- LNPs sizes (nm) after stabilization	mRNA- LNPs PDI values after stabilization	Accepted criteria (size should be stable)
40 – 60	50.73±2.55	0.431±0.003	55.09±2.44	0.430±0.004	stable*
80 – 100	86.31±1.76	0.151±0.001	88.98±1.70	0.114±0.016	stable
100 – 120	112.47±2.23	0.044±0.004	113.41±4.29	0.029±0.031	stable
120 – 140	125.85±0.92	0.050±0.007	127.04±1.32	0.048±0.004	stable
140 – 160	144.87±1.61	0.189±0.005	144.60±0.80	0.199±0.057	stable
160 – 180	161.13±1.41	0.124±0.004	162.41±0.56	0.147±0.024	stable

*, Particle size matched but PDI value failed

Appendix A-Supplementary information

Supplementary Table 12. Reproducibility and scalability of mRNA-LNPs production

Batch size	Batch	Replicates	LNPs size (nm) after formulation	Average LNPs size (nm) after formulation	Average LNPs size (nm) after formulation (batch 01,02 and 03)	PDI Values after formulation	Average PDI values after formulation	Average PDI values after formulation (batch 01,02 and 03)
1× (10 ml)	Batch 01	R01	70.26	73.43±2.46	72.00±1.27	0.151	0.160±0.006	0.143±0.025
		R02	76.25			0.163		
		R03	73.78			0.161		
		R04	73.43			0.165		
	Batch 02	R01	68.26	70.99±2.76		0.151	0.154±0.006	
		R02	69.14			0.163		
		R03	74.16			0.153		
		R04	72.4			0.149		
	Batch 03	R01	72.16	71.57±1.83		0.116	0.114±0.004	
		R02	69.56			0.11		
		R03	73.81			0.119		
		R04	70.75			0.111		
10× (100 ml)	Batch 01	R01	81.16	79.49±1.14	78.82±0.15	0.158	0.167±0.008	0.153±0.011
		R02	78.67			0.176		
		R03	79.25			0.172		
		R04	78.88			0.162		
	Batch 02	R01	76.74	79.91±2.90		0.147	0.152±0.009	
		R02	78.17			0.142		
		R03	82.19			0.158		
		R04	82.54			0.161		
	Batch 03	R01	74.81	77.07±2.13		0.138	0.145±0.006	
		R02	79.51			0.149		
		R03	78.1			0.142		
		R04	75.86			0.151		
100× (1000 ml)	Batch 01	R01	74.16	75.80±2.26	74.91±0.80	0.136	0.132±0.003	0.120±0.034
		R02	73.61			0.131		
		R03	78.19			0.128		
		R04	77.24			0.133		
	Batch 02	R01	72.56	74.67±2.11		0.142	0.146±0.006	
		R02	76.59			0.149		
		R03	73.15			0.153		
		R04	76.38			0.14		
	Batch 03	R01	77.19	74.26±4.91		0.101	0.081±0.015	
		R02	72.16			0.083		
		R03	68.41			0.072		
		R04	79.28			0.068		

N.B.: Acceptance criteria (LNP particle size: 60-80 nm, PDI: ≤ 0.200).

Appendix A-Supplementary information

Supplementary Table 13. Encapsulation efficiency of developmental batch and scaled up batches (4 replicates from each batch)

Batch size	Batch	Replicates	Percentage of encapsulation	Average percentage of encapsulation	Average Percentage of encapsulation (batch 01,02 and 03)	Copy no. of encapsulated mRNA ($\times 10^8$)	Average copy no. of encapsulated mRNA ($\times 10^8$)	Average copy no. of encapsulated mRNA ($\times 10^8$) (batch 01,02 and 03)
1× (10 ml)	Batch 01	R01	91	92.50±1.29	93.33±1.83	3.50	3.78±0.30	3.92±0.27
		R02	93			4.20		
		R03	94			3.70		
		R04	92			3.70		
	Batch 02	R01	92	93.75±2.06		3.90	4.05±0.28	
		R02	96			4.24		
		R03	95			4.32		
		R04	92			3.73		
	Batch 03	R01	93	93.75±2.22		3.81	3.94±0.21	
		R02	95			4.18		
		R03	91			3.72		
		R04	96			4.05		
10× (100 ml)	Batch 01	R01	93	92.00±3.11	93.92±2.23	4.15	4.02±0.27	4.09±0.31
		R02	98			4.32		
		R03	91			3.86		
		R04	92			3.73		
	Batch 02	R01	92	94.25±2.22		4.40	4.01±0.34	
		R02	95			3.62		
		R03	97			4.15		
		R04	93			3.86		
	Batch 03	R01	93	94.00±1.83		4.60	4.26±0.32	
		R02	96			4.40		
		R03	92			4.19		
		R04	95			3.84		
100× (1000 ml)	Batch 01	R01	96	93.50±2.08	93.44±1.94	3.60	4.01±0.28	3.99±0.25
		R02	91			4.05		
		R03	94			4.16		
		R04	93			4.22		
	Batch 02	R01	92	92.00±1.41		3.83	4.07±0.24	
		R02	94			4.32		
		R03	91			3.90		
		R04	91			4.21		
	Batch 03	R01	93	95.00±1.41		3.69	3.86±0.24	
		R02	95			4.21		
		R03	96			3.81		
		R04	96			3.71		

N.B.: Acceptance criteria of encapsulation efficiency (EE≥ 90±5%) and copy no. (≥ 3.75×10^8)

Appendix A-Supplementary information

Supplementary Table 14. Yield of developmental batch and scaled up batches

Batch size	Batch	Theoretical mRNA quantity	Expected dose volume (ml)	Percentage of encapsulation efficiency (EE)	Final dose volume (ml)	% Yield	%Yield (Average)
1× (10 ml)	Batch 01	1 mg	10	92.50	8.5	78.63	
	Batch 02	1 mg	10	93.75	9.2	86.25	82.15±3.85
	Batch 03	1 mg	10	93.75	8.7	81.56	
10× (100 ml)	Batch 01	10 mg	100	92.00	95.0	87.40	
	Batch 02	10 mg	100	94.25	93.0	87.65	88.12±1.03
	Batch 03	10 mg	100	94.00	95.0	89.30	
100× (1000 ml)	Batch 01	100 mg	1000	93.50	945	88.36	
	Batch 02	100 mg	1000	92.00	925	85.10	87.27±1.88
	Batch 03	100 mg	1000	95.00	930	88.35	

N.B.: Acceptance criteria: %Yield should be ≥ 80. [% Yield = (final dose volume ×%EE/ expected dose volume)]
